# Supplementary material for: Conditional chemoconnectomics (cCCTomics) as a strategy for efficient and conditional targeting of chemical transmission
Source: eLife. 2024 Apr 30;12:RP91927. doi: 10.7554/eLife.91927 (PMC11060718; doi:10.7554/eLife.91927)
Supplement: Figure 3—source data 3. [file elife-91927-fig3-data3.zip › Figure 3 source data3 part 2.zip!/Figure 3-Source data 3-part2/1053-1-CRISPResso_Report_mpeqg9da.zip!/CRISPResso_on_1053_2/9.Alleles_frequency_table_around_sgRNA_ggagcggaatctacaaaacg.pdf]

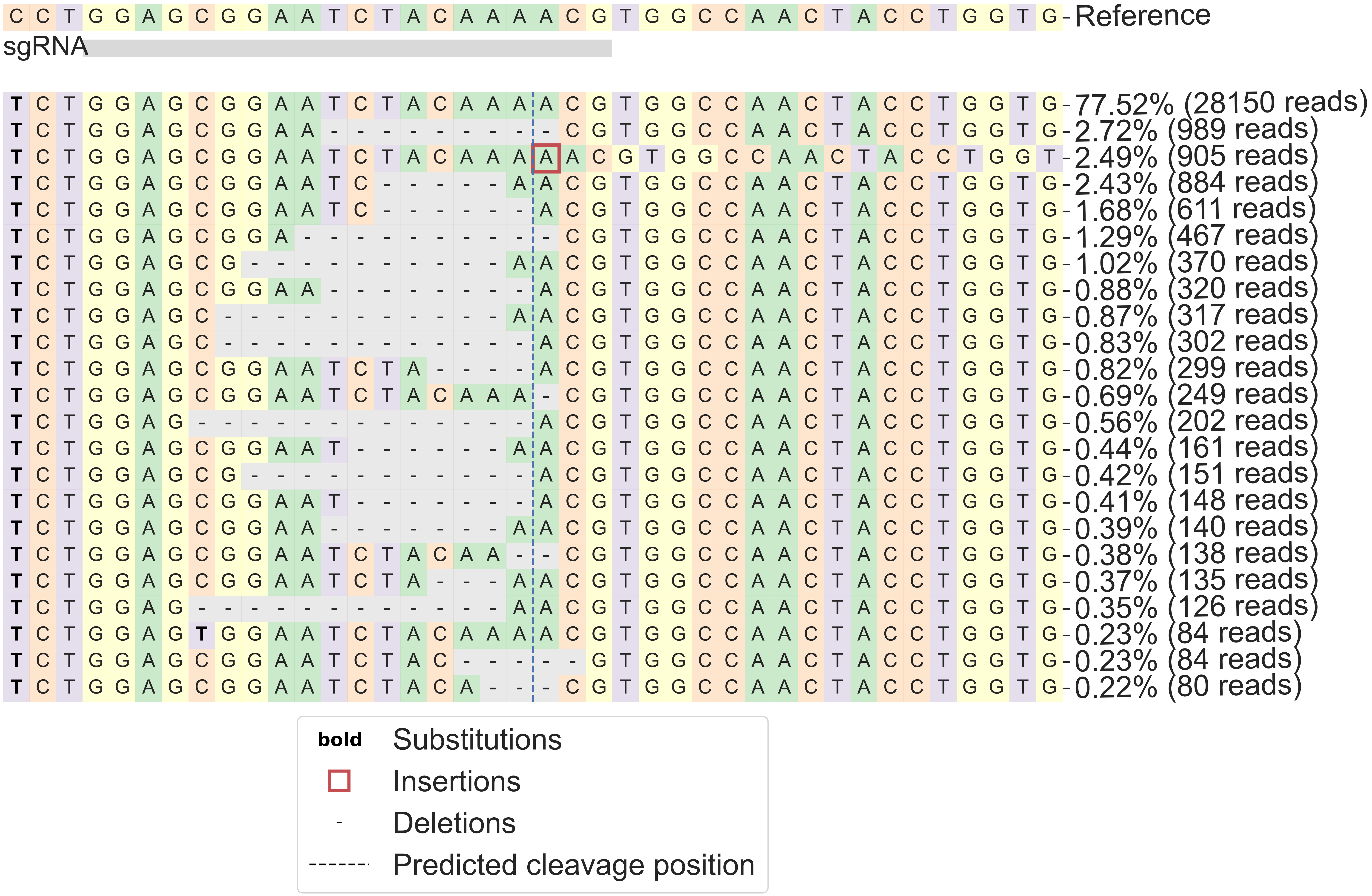

C C T G G A G C G G A A T C T A C A A A A C G T G G C C A A C T A C C T G G T G - Reference
sgRNA
77.52% (28150 reads)
2.72% (989 reads)
2.49% (905 reads)
2.43% (884 reads)
1.68% (611 reads)
1.29% (467 reads)
1.02% (370 reads)
0.88% (320 reads)
0.87% (317 reads)
0.83% (302 reads)
0.82% (299 reads)
0.69% (249 reads)
0.56% (202 reads)
0.44% (161 reads)
0.42% (151 reads)
0.41% (148 reads)
0.39% (140 reads)
0.38% (138 reads)
0.37% (135 reads)
0.35% (126 reads)
0.23% (84 reads)
0.23% (84 reads)
0.22% (80 reads)
bold Substitutions
Insertions
Deletions
Predicted cleavage position
